# Chemical Priming by Neonicotinoids Unveils *CaNEN4* as a Susceptibility Gene Against *Phytophthora capsici* in Pepper

**DOI:** 10.1111/mpp.70242

**Published:** 2026-03-19

**Authors:** Geng Meng, Shujia Wang, Yiheng Hou, Wenqing Li, Shiwei Yang, Tianhao Ge, Chenxue Song, Peng Liu, Wenyi Yang, Gonglian Pang, Zhiqi Jia, Jianbin Hu, Chengwei Li, Yawen Shen, Kaile Sun

**Affiliations:** ^1^ College of Horticulture Henan Agricultural University Zhengzhou China; ^2^ International Joint Laboratory of Henan Horticultural Crop Biology Zhengzhou China; ^3^ School of Agricultural Sciences Zhengzhou University Zhengzhou China

**Keywords:** *Capsicum frutescens*, chemical priming, *Phytophthora capsici*, ROS homeostasis, susceptibility gene

## Abstract

The oomycete *Phytophthora capsici* causes Phytophthora blight, a major constraint on global pepper production. Our previous observations indicated that pretreating plants with thiamethoxam (TMX) and imidacloprid (IMI) could reduce the incidence of pepper blight, but the underlying mechanisms remained unclear. Here, we investigated how TMX and IMI induced resistance in pepper ( *Capsicum frutescens* ) against *P. capsici*. Both in vitro and in vivo assays demonstrated that TMX and IMI suppressed disease, not by directly impairing pathogen virulence but by inducing systemic resistance in susceptible (Cusheng L09) and resistant (Cusheng 356) pepper cultivars. Split‐plant systemic resistance assays showed that TMX/IMI‐primed plants developed smaller lesions in both treated and untreated leaves following *P. capsici* infection. Foliar application of TMX and IMI effectively alleviated disease severity, with IMI showing superior efficacy in attenuating reactive oxygen species (ROS) accumulation, and TMX/IMI priming concomitantly altering the activities of ROS‐scavenging enzymes under pathogen challenge. Reverse transcription‐quantitative PCR analysis revealed time‐dependent changes in defence gene expression, and whole‐genome transcriptome profiling highlighted temporal reprogramming of pathogenesis‐related genes. Further functional validation identified *CaNEN4* as a susceptibility factor. Collectively, our findings reveal that IMI/TMX primes pepper plants with systemic resistance by modulating ROS homeostasis, defence gene expression, and susceptibility gene function, offering novel insights into chemical‐induced plant immunity and genetic targets for durable blight resistance in crops.

## Graphical abstract

IMI/TMX priming induces resistance to *Phytophthora capsici* by balancing ROS and suppressing RNA‐seq‐identified susceptibility genes, including *CaNEN4*, for sustainable management of pepper Phytophthora blight.

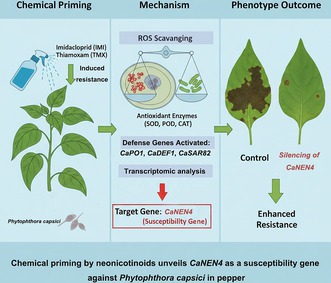

## 1 Introduction

Pepper (*Capsicum* spp.), a globally significant vegetable crop with multifaceted uses as food, spice, medicine and ornamental plant. It is rich in bioactive compounds, particularly high levels of vitamin C and vitamin E, contributing to its nutritional and health‐promoting qualities (He et al. 2018; Wang, Yang, et al. 2024; Gai et al. 2020; Li, Jia, et al. 2024). Among *Capsicum* species, *Capsicum frutescens* , closely related to *C. chinense* , is distinguished by its small, upright fruits with high pungency (Baral and Bosland 2004; Jarret et al. 2007).

Pepper cultivation faces severe threats from pepper blight caused by the soil‐borne oomycete pathogen *Phytophthora capsici* (Akgül and Mirik 2008; Moreira‐Morrillo et al. 2022). *P. capsici* causes root rot, stem necrosis, foliar blight, and fruit rot across all growth stages (Esfahani et al. 2014), with disease severity increasing under warm (25°C–30°C) and humid conditions. This pathogen can survive as oospores or chlamydospores in soil and plants for up to 3 years, and it produces sporangia within 2–3 days under optimal humidity (> 70%), resulting in repeated infection cycles within a single growing season (Santos et al. 2023). *P. capsici* causes annual yield losses of nearly $100 million, making it the fifth most destructive oomycete pathogen worldwide (Kamoun et al. 2015; Moreira‐Morrillo et al. 2022).

Durable resistance is critical for sustainable pepper production, with genetic resistance being a promising strategy. Recent studies on *Capsicum annuum* (bell pepper) have made progress in combating root and basal rot disease caused by *P. capsici* (Bagheri et al. 2021). Seven novel candidate genes were found to be upregulated in *P. capsici*‐infected peppers by Bagheri et al. (2020), laying a foundation for further gene functional verification and understanding the regulatory mechanism of pepper resistance to *P. capsici*. The resistant germplasm *Capsicum annuum* cultivar CM334 exhibits polygenic resistance through a major quantitative trait locus (QTL) on chromosome P5 (Phyto5.2) (Quirin et al. 2005; Lefebvre and Palloix 1996). Additionally, PI201234 has a distinct resistance locus separable from Phyto5.2 (Wang et al. 2016). While molecular markers (e.g., SCAR OpD04.717) assist breeding, marker‐trait correlations are often unstable due to pathogen variability and race specificity (Ortega et al. 1991; Sy et al. 2005).

Susceptibility to *P. capsici* can arise from either the absence of resistance (*R*) genes or the presence of susceptibility (*S*) genes. Unlike *R* genes, *S* genes like *MLO* confer durable, broad‐spectrum resistance when functionally disrupted. This strategy has been successfully applied in several crops: *MLO* knockouts confer powdery mildew resistance in barley, wheat, and tomato (Bai et al. 2008; Pessina, Angeli, et al. 2016; Pessina, Lenzi, et al. 2016; Li et al. 2022). Reeves et al. (2013) identified a gene in pepper, named *Ipcr* (inhibitor of six *Phytophthora* spp. resistance), that negatively regulates disease resistance. Traditional breeding strategies have largely focused on pyramiding *R* genes; however, this approach often suffers from race‐specific limitations. Targeting *S* genes, especially via CRISPR‐mediated editing, offers a promising alternative for durable, broad‐spectrum resistance (Zhang et al. 2021).

Neonicotinoid insecticides, including imidacloprid (IMI) and thiamethoxam (TMX), are widely used globally, targeting insect nicotinic receptors while exhibiting low vertebrate toxicity (Mullins 1993; Maienfisch et al. 2001; Huang et al. 2019; Yan et al. 2023; Li, Zhu, et al. 2024; Cai et al. 2022). The biological activity of TMX and IMI is largely determined by their oxidative metabolites: IMI degrades into 6‐chloropyridinyl‐3‐carboxylic acid (CPA), while TMX metabolises to 2‐chlorothiazolyl‐5‐carboxylic acid (CTA). They exhibit structural homology with salicylic acid (SA), a phytohormone central to systemic acquired resistance (SAR), and may functionally mimic SA signalling pathways (Ford et al. 2010; Vlot et al. 2009). Analysing whether they induce plant resistance could facilitate the identification of new genes related to disease resistance.

Identification of different cultivars based on disease resistance is key for the selection and development of highly disease‐resistant genotypes (Alizadeh‐Moghaddam et al. 2024). Here, systematic screening of 16 germplasm resources identified a resistant cultivar and a highly susceptible cultivar, establishing a robust system for comparative resistance analysis. Foliar application of neonicotinoids enhanced host resistance, with physiological profiling revealing reduced reactive oxygen species (ROS) bursts and upregulated defence regulators. Transcriptome‐wide interrogation identified eight *S* gene candidates, with *CaNEN4* emerging as a novel negative regulator. These findings provide a promising foundation for breeding more durable, disease‐resistant pepper cultivars.

## 2 Results

### 2.1 Evaluation of *P. capsici* Resistance in Pepper Germplasm

**FIGURE 1:**
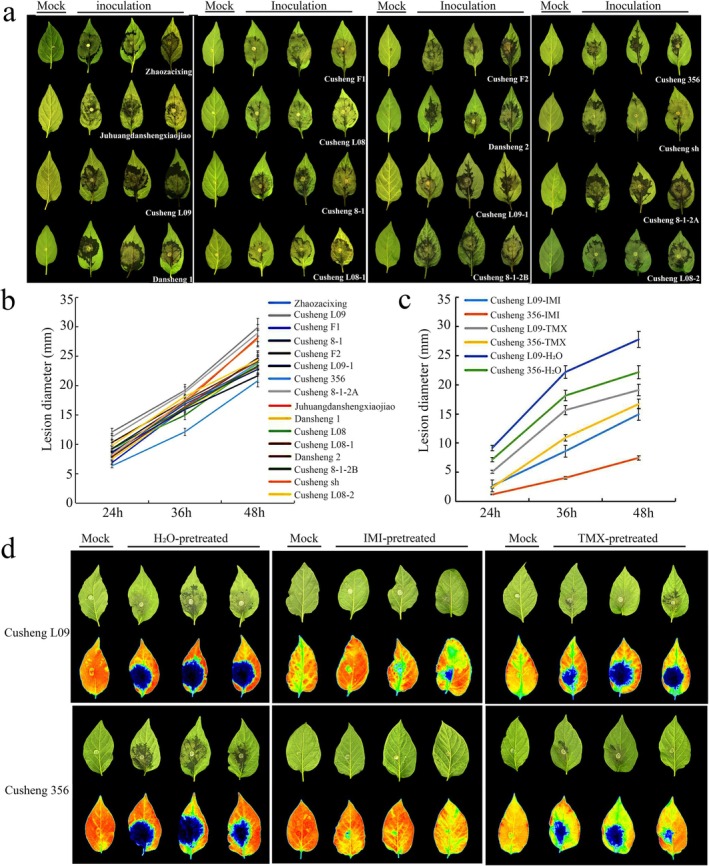
Assessment of *Phytophthora capsici* resistance in pepper cultivars with or without exogenous imidacloprid (IMI) or thiamethoxam (TMX) pretreatment. (a) Disease symptoms of different pepper cultivars inoculated with *P. capsici* at 48 h post‐inoculation (hpi). (b) Lesion diameters measured at different time points after *P. capsici* inoculation. Data are shown as means ± SD (*n* = 3). (c) Lesion diameters accessed at different time points in resistant (Cusheng 356) and susceptible (Cusheng L09) cultivars pretreated with IMI or TMX and inoculated with *P. capsici*. (d) Disease symptoms of pepper leaves pretreated with water or IMI/TMX at 24 hpi. The upper panel displays the disease phenotypes, while the lower panel shows the chlorophyll fluorescence intensities for each cultivar.

Using the in vitro leaf inoculation method, we evaluated the *P. capsici*‐induced pepper blight disease resistance among 16 pepper cultivars (Figure 1a; Figure S1, Table S1). Based on the established resistance classification standards, Cusheng L09‐1 and Cusheng 356 were identified as low resistant, with Cusheng 356 having the lowest disease index, and the remaining materials were classified as susceptible, with Cusheng L09 exhibiting the highest disease index (Figure 1b; Table S2).

### 2.2 Assessment of IMI‐ and TMX‐Induced Pepper Blight Resistance

Our previous observations indicated that plants pretreated with IMI and TMX could reduce the incidence of pepper blight. To further clarify the mechanism, we conducted an in vitro leaf inoculation assay on susceptible cultivar Cusheng L09 and resistant cultivar Cusheng 356. Plants were pretreated three times within 1 week with 1 mM IMI or TMX (pesticide‐grade), while control plants received sterile water, before pathogen inoculation. The lesion diameters were measured at 24, 36, and 48 h post‐inoculation (hpi) (Figure 1c,d). At 48 hpi, IMI and TMX pretreatments reduced the lesion diameters by approximately 46% and 31% in Cusheng L09, respectively, compared to the control. In Cusheng 356, the lesion diameters were reduced by 66% and 25% under IMI or TMX treatment, respectively. These results indicated that IMI or TMX significantly enhance pepper resistance to *P. capsici*, particularly in IMI‐pretreated plants.

### 2.3 Effects of IMI and TMX on *P. capsici* Viability and Pathogenicity

**FIGURE 2:**
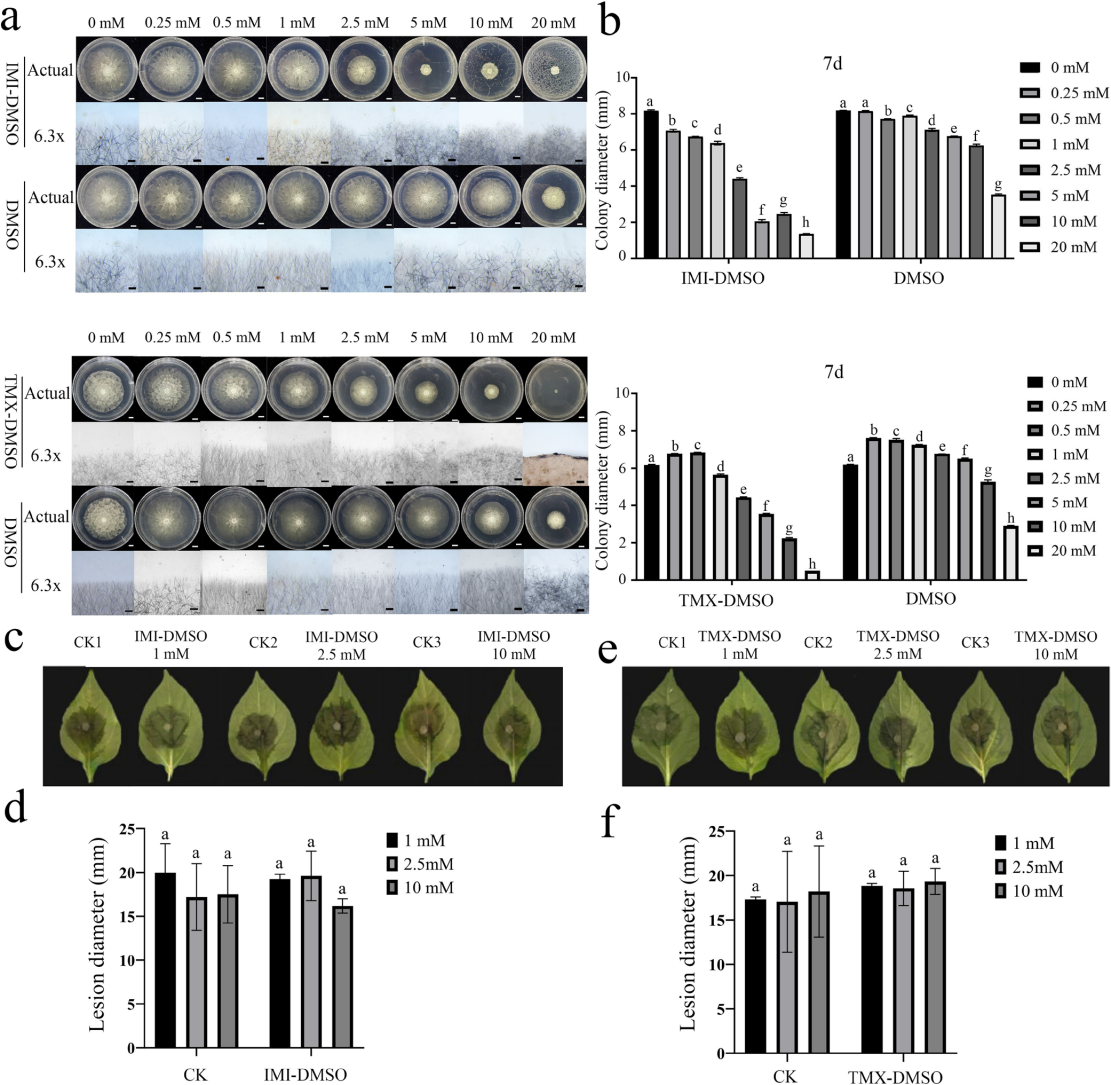
The effects of exogenous application of imidacloprid (IMI) and thiamethoxam (TMX) on the growth and pathogenicity of *Phytophthora capsici*. (a) Changes in colony diameter on the seventh day following reagent‐grade IMI and TMX treatments. IMI and TMX were dissolved in dimethylsulphoxide (DMSO) and diluted in water (IMI‐DMSO, TMX‐DMSO); controls received equivalent DMSO concentrations (DMSO). (b) Colony diameters of *P. capsici* treated with different concentrations of IMI or TMX. (c, d) *P. capsici* were pre‐exposed to IMI (1, 2.5 or 10 mM) or to mock DMSO medium (CK) and then inoculated onto leaves of susceptible pepper (cv. Cusheng L09) for pathogenicity assessment. Representative lesion symptoms at 48 h post‐inoculation (hpi) (c) and lesion diameter quantification (d). (e,f) Lesion symptoms (e) and lesion diameters (f) of Cusheng L09 leaves inoculated with *P. capsici* pre‐exposed to TMX‐containing medium at 48 hpi. Different letters above bars indicate significant differences among concentrations within the same treatment group as determined by one‐way ANOVA followed by LSD multiple comparisons (*p* < 0.05).

Considering the residues of IMI and TMX on leaves may influence *P. capsici* growth, we cultured *P. capsici* on growth media containing varying concentrations of each compound. To account for the influence of formulation additives, both pesticide‐grade (TMX‐H_2_O, IMI‐H_2_O; 25% and 70% active ingredient, respectively) dissolved in water and reagent‐grade compounds (TMX‐DMSO and IMI‐DMSO) dissolved in dimethyl sulphoxide (DMSO) and diluted in water were tested in parallel. Colony growth was continuously monitored over 7 days (Figures S2 and S3). Both IMI and TMX inhibited *P. capsici* in a concentration‐dependent manner, with 2.5 mM reducing mycelial growth by 75%–78% and 10 mM exceeding 90% inhibition (Tables S4 and S5). Hyphal morphology changed from compact to irregular and highly branched at high concentrations of reagent‐grade IMI or TMX (1–20 mM), eventually leading to complete growth suppression (Figure 2a,b).

To assess their effects on *P. capsici* pathogenicity, mycelial plugs from growth media with or without TMX/IMI were inoculated onto detached susceptible cultivar Cusheng L09 pepper leaves. Lesion diameters at 48 hpi were not significantly different across treatments (Figure 2c–f), demonstrating the growth‐inhibitory effects of TMX and IMI on *P. capsici* are transient and reversible with no lasting impact on *P. capsici* pathogenicity. This suggests that the observed disease suppression in planta is probably mediated by enhanced host resistance mechanisms rather than the direct antifungal activity of the insecticides.

### 2.4 TMX and IMI Induced Systemic Resistance to *P. capsici*

**FIGURE 3:**
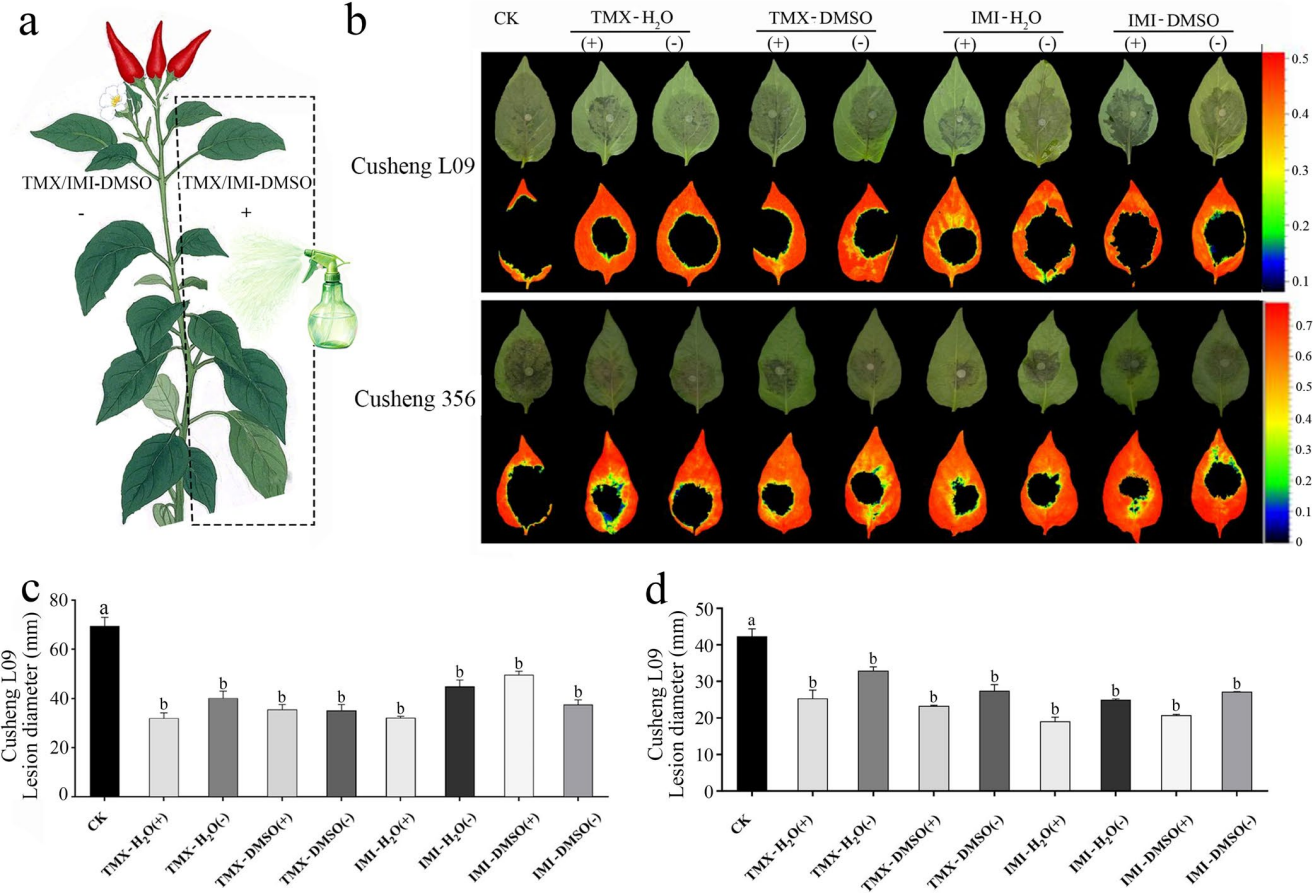
Thiamethoxam (TMX) and imidacloprid (IMI) can induce systemic resistance in peppers. (a) Schematic diagram explaining the split‐plant treatment. TMX‐H_2_O or IMI‐H_2_O refers to pesticide‐grade TMX or IMI dissolved in water; TMX‐DMSO or IMI‐DMSO refers to reagent‐grade TMX or IMI dissolved in dimethyl sulphoxide (DMSO) and diluted in water. (+) and (−) indicate leaves of the same plant pretreated or not pretreated, respectively, with TMX and IMI. (b) Disease symptoms on Cusheng L09 (susceptible cultivar) and Cusheng 356 (resistant cultivar) at 72 h post‐inoculation (hpi) on the same plant with or without TMX and IMI pretreatment (reagent‐grade or pesticide‐grade) or control plants received no pesticide (CK). (c, d) Lesion diameters at 72 hpi on Cusheng L09 and Cusheng 356, respectively. Different letters above bars indicate significant differences between CK and different treatments as determined by one‐way ANOVA (*p* < 0.05).

To elucidate whether TMX and IMI enhance pepper resistance to *P. capsici* through host defence priming, a split‐plant systemic resistance assay was conducted on Cusheng L09 and Cusheng 356 plants. In each plant, four leaves were selected, with one side treated with 1 mM IMI or TMX (marked with ‘+’) and the opposite side mock‐treated with sterile water or 0.2% DMSO as a solvent control (marked with ‘–’) (Figure 3a). Lesion area and fluorescence intensity measurements further confirmed that IMI‐ or TMX‐treated plants, especially the resistant cultivar Cusheng 356, exhibited smaller disease symptoms on both treated and untreated leaves compared to control plants (Figure 3b). In susceptible cultivar Cusheng L09 at 72 hpi, both reagent‐ and pesticide‐grade TMX reduced lesion diameters by 49%–53% in treated leaves (TMX+) and 42%–49% in untreated leaves (TMX–), respectively. IMI reduced lesions by 28%–53% in treated leaves (IMI+) and 35%–46% in untreated leaves (IMI–) (Figure 3c). In resistant cultivar Cusheng 356, TMX yielded 40%–45% (TMX+) and 22%–35% (TMX–) reductions, while IMI treatment suppressed lesions by 50%–55% (IMI+) and 35%–41% (IMI–) (Figure 3d). These results suggest that both TMX and IMI induce systemic resistance in pepper, rather than acting through local antifungal activity.

### 2.5 TMX/IMI Induced Systemic Antioxidant Responses in Pepper During *P. capsici* Infection

**FIGURE 4:**
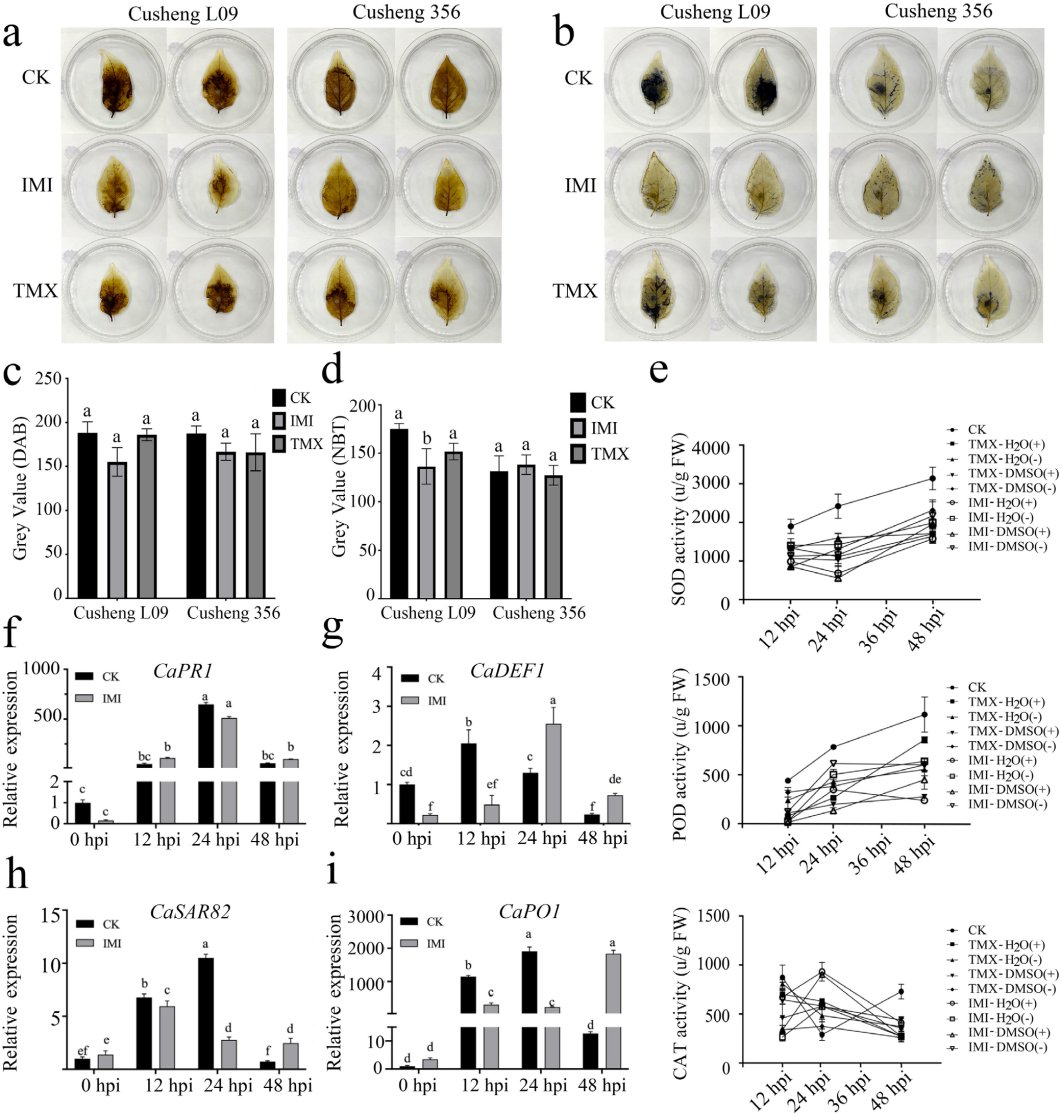
Thiamethoxam (TMX) and imidacloprid (IMI) pretreatment reduce reactive oxygen species (ROS) accumulation and activate antioxidant responses in pepper during *Phytophthora capsici* infection. (a–d) 3,3′‐diaminobenzidine (DAB) and nitroblue tetrazolium (NBT) staining at 48 h post‐inoculation (hpi) in Cusheng 356 and Cusheng L09. (a, b) Representative images of DAB‐ and NBT‐stained leaves pretreated with TMX or IMI and inoculated with *P. capsici* using the split‐plant system. (c, d) Quantification of staining intensities. Different letters above bars indicate significant difference by one‐way ANOVA (*p* < 0.05). (e) Antioxidant enzyme activities (superoxide dismutase [SOD], peroxidase [POD], and catalase [CAT]) in leaves pretreated with (+) or without (−) TMX‐ or IMI plants at 48 hpi in the split‐plant assay, leaves from plants pretreated with TMX or IMI (+) or not pretreated (−); CK, plants received no pesticide. (f–i) Relative expression of pathogenesis‐related genes (*CaPR1*, *CaDEF1*, *CaSAR82* and *CaPO1*) analysed by reverse transcription‐quantitative PCR. Gene expression was analysed by two‐way ANOVA followed by LSD multiple Fisher's comparisons test. Different letters indicate significant differences (*p* < 0.05).

To investigate whether IMI and TMX affect ROS accumulation in pepper leaves following *P. capsici* inoculation, 3,3′‐diaminobenzidine (DAB) and nitroblue tetrazolium (NBT) staining assays were performed. DAB staining detects hydrogen peroxide (H_2_O_2_), while NBT staining detects superoxide anions (O_2_^−^). In the split‐plant system, at 48 hpi, the IMI‐ or TMX‐primed susceptible Cusheng L09 leaves (IMI/TMX+) displayed reduced DAB and NBT staining relative to the water mock control (Figure 4a–d). By contrast, in the resistant cultivar Cusheng 356, no statistically significant differences were detected after IMI/TMX pretreatment (Figure 4a–d).

To assess whether TMX and IMI influence antioxidant responses, ROS‐scavenging enzyme activities were quantified using the abovementioned split‐plant system. At 12–48 hpi, superoxide dismutase (SOD) and peroxidase (POD) activities in control plants increased markedly, reflecting strong oxidative stress, whereas both activities were lower in IMI‐ and TMX‐treated plants (IMI/TMX+). Notably, untreated contralateral (IMI/TMX–) leaves also exhibited reduced SOD and POD activities compared to controls (CK) upon *P. capsici* infection, indicating a systemic effect of the compounds. Catalase (CAT) activity was higher in CK at 48 hpi but showed minimal differences between IMI/TMX treatments at 12 and 24 hpi (Figure 4e), indicating reduced ROS accumulation in both insecticide‐treated and untreated leaves compared to CK.

Reverse transcription‐quantitative PCR (RT‐qPCR) assays further identified that several pathogenesis‐related (PR) genes were differentially expressed in IMI‐pretreated Cusheng 356 leaves, followed by *P. capsici* infection. *CaPR1*, a marker of salicylic acid (SA)‐mediated defence, showed no significant differences between IMI‐primed and control plants at all time points (Figure 4f). *CaDEF1*, involved in methyl jasmonate (MeJA)‐mediated signalling, was expressed higher in controls at 0–12 hpi but significantly upregulated in IMI‐treated leaves at 24–48 hpi (Figure 4g). For *CaSAR82*, part of SA‐mediated signalling, its expression level was lower in the IMI‐treated leaves compared to controls at 12 and 24 hpi and increased significantly by 48 hpi (Figure 4h). *CaPO1*, linked to H_2_O_2_ accumulation and peroxidase activity during programmed cell death, showed no difference at 0 hpi, lower expression in IMI‐treated leaves at 12–24 hpi, and higher expression at 48 hpi compared to controls (Figure 4i).

### 2.6 Transcriptomic Profiling of IMI‐Primed Pepper Highlights Defence Pathways Against *P. capsici*

**FIGURE 5:**
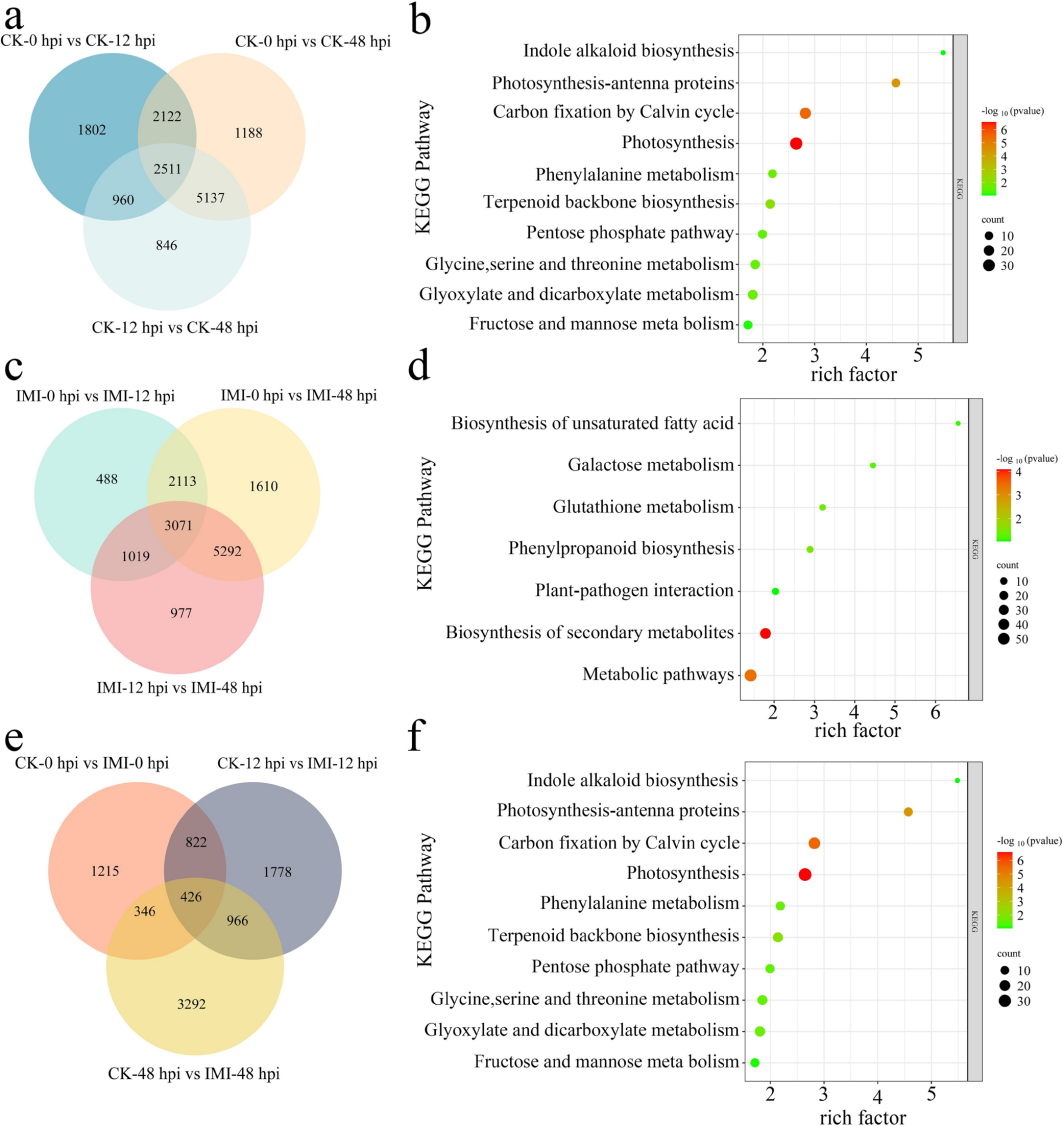
Transcriptomic data analysis at different time points after inoculation of pepper plants with *Phytophthora capsici* in the control and imidacloprid (IMI)‐primed groups. (a, b) Venn analysis and KEGG analysis of differentially expressed genes (DEGs) in the control group at different post‐inoculation time points. (c, d) Venn analysis and KEGG analysis of DEGs in the IMI‐primed group at different post‐inoculation time points. (e, f) Venn analysis and KEGG analysis of DEGs between the control and IMI‐primed groups at different post‐inoculation time points.

To further investigate the molecular mechanisms underlying pepper resistance, transcriptomic profiling of IMI‐primed Cusheng 356 pepper plants was performed to reveal temporal and treatment‐specific responses to *P. capsici* infection. In control plants at 0, 12 and 48 hpi, 2511 consistently differentially expressed genes (DEGs) were identified, with KEGG enrichment highlighting photosynthesis and carbon fixation by Calvin circle, and phenylalanine pathways (Figure 5a–b). In IMI‐primed plants, time‐course analysis revealed 3071 DEGs predominantly enriched in biosynthesis of primary (e.g., galactose and glutathione), secondary metabolites, and plant–pathogen interaction (Figure 5c–d). Comparison between primed and control plants identified 426 DEGs, with significant enrichment in photosynthesis pathways (Figure 5e–f). RT‐qPCR validation of eight randomly selected DEGs showed expression patterns largely consistent with RNA‐Seq data, confirming the reliability of the transcriptomic results (Figure S4).

### 2.7 *CaNEN4* Functions as a Susceptibility Gene

**FIGURE 6:**
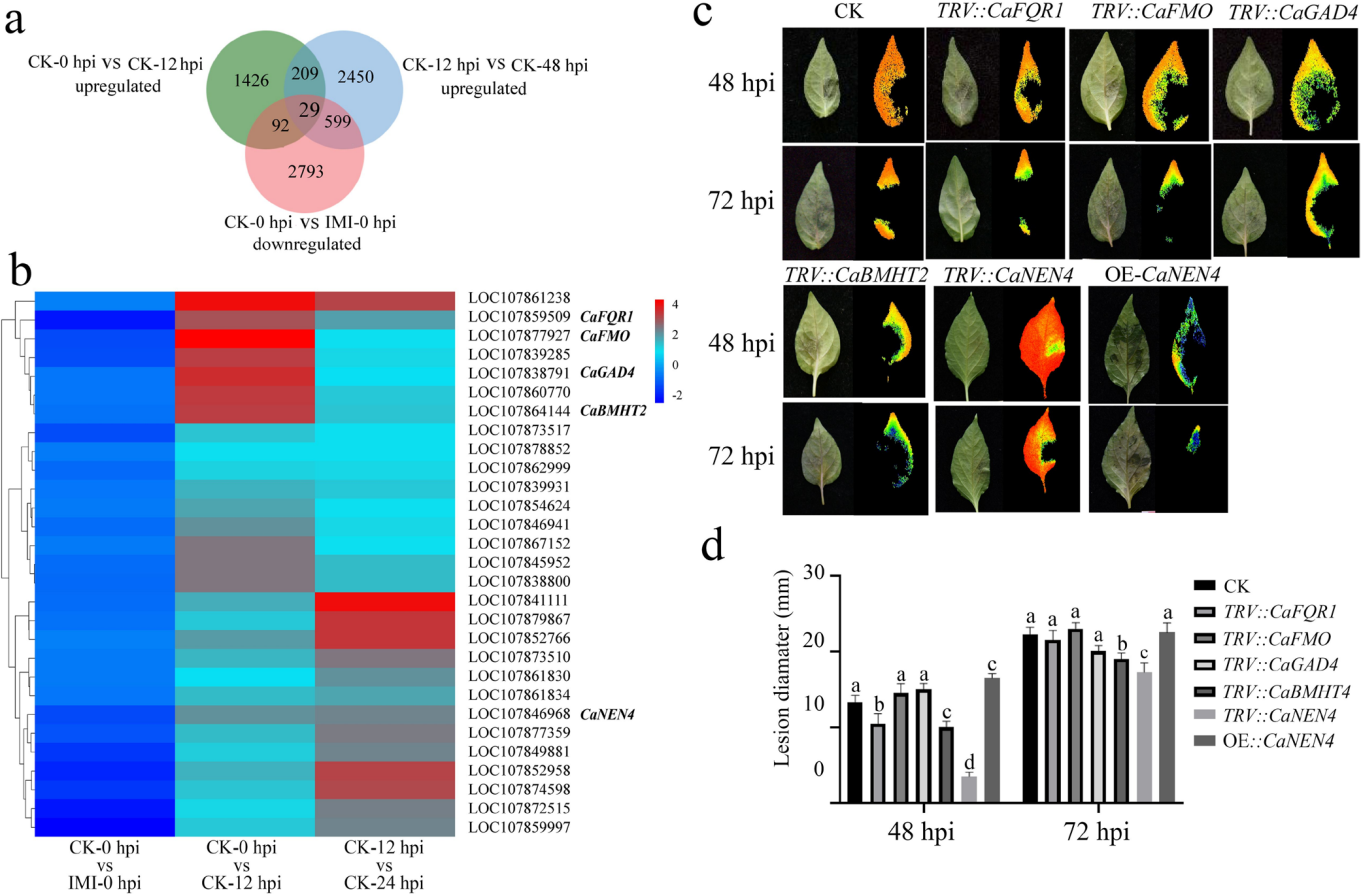
Identification of candidate susceptibility genes. (a) Venn diagram of candidate susceptibility genes. (b) Heatmap of the expression patterns of susceptibility genes. (c) Phenotypes of candidate susceptibility genes after transient silencing and overexpression at 48 and 72 h post‐inoculation (hpi). (d) Lesion diameter of transient silencing and overexpression plants. Within each time point, differences were analysed by one‐way ANOVA with Fisher's LSD (*p* < 0.05); different letters indicate significant differences.

To identify potential susceptibility genes, we conducted Venn analysis on three sets of DEGs: genes downregulated in control compared to IMI‐treated plants at 0 hpi, genes upregulated in control plants from 0 to 12 hpi, and genes upregulated in control plants from 12 to 48 hpi. This analysis revealed 29 common DEGs to all comparisons (Figure 6a). We further performed virus‐induced gene silencing (VIGS) on five DEGs with the highest expression fold‐changes. Upon *P. capsici* infection, silencing of *NAC45/86‐dependent exonuclease‐domain protein 4* (*CaNEN4*) and *betaine‐homocysteine methyltransferase‐2* (*CaBMHT2*) significantly enhanced resistance, resulting in a smaller lesion area at 48 and 72 hpi compared to controls, whereas silencing of the other candidates showed no visible phenotypic change. Additionally, transient overexpression of *CaNEN4* in pepper plants increased disease susceptibility, with larger *P. capsici*‐induced necrosis in control plants at 48 hpi. Together, these results demonstrate that CaNEN4 acts as a potential susceptibility factor contributing to *P. capsici* infection in pepper.

## 3 Discussion

*Phytophthora capsici* severely limits global pepper production, as a strong resistance cultivar is lacking due to the genetic uniformity of cultivated *C. frutescens* and the pathogen's high race diversity. Fungicide‐based control is increasingly ineffective owing to *P. capsici*'s reduced sensitivity and rapid resistance development.

This study demonstrates that the neonicotinoids TMX and IMI can enhance pepper resistance to *P. capsici* through activating plant defence responses. Notably, when TMX or IMI was applied to only one side of a plant, both treated and untreated leaves showed enhanced resistance, indicating systemically activated defence independent of direct chemical contact. This finding aligns with earlier reports of neonicotinoid‐induced SAR in citrus (Graham and Myers 2013), although the underlying immunological mechanisms remain to be clarified.

ROS play dual roles in plant defence, functioning as both antimicrobial agents and secondary messengers that amplify immune signalling (Wang et al. 2023; Zhang et al. 2020; Du et al. 2023; Li et al. 2025; Xiang et al. 2025). Studies in wheat demonstrated rapid ROS accumulation during *Puccinia striiformis* invasion triggers localised hypersensitive cell death, effectively restricting pathogen spread (Wang, Fan, et al. 2022). Similarly, transgenic tomato overexpressing *SlRIPK* exhibit pathogen‐induced ROS bursts associated with broad‐spectrum immunity (Wang, Li, et al. 2022). Interestingly, our findings showed that IMI‐ and TMX‐treated pepper plants displayed enhanced resistance to *P. capsici*, a pattern consistent with the findings in rice *OsERF65* knockout, which displays enhanced resistance to sheath blight while maintaining reduced H_2_O_2_ content compared to wild‐type (WT) plants (Xie et al. 2023). Correspondingly, SOD and POD activities were significantly lower in TMX‐ and IMI‐treated pepper plants than in controls, whereas CAT activity was transiently elevated at 24 hpi before declining. These results contrast with earlier studies reporting a positive correlation between POD activity and disease resistance, such as in rice bacterial blight, tomato and potato early blight (Wu et al. 1996; Alizadeh‐Moghaddam et al. 2020; Peymani et al. 2022) and pepper cultivars differing in *P. capsici* resistance (Zhang et al. 2013). After *P. capsici* inoculation, both *CaSBP12* transgenic lines and WT pepper plants showed an increase in POD activity, while CAT activity initially rose, then decreased (Zhang et al. 2019). Given that SOD catalyses the conversion of O_2_^−^ to H_2_O_2_, while POD and CAT decompose H_2_O_2_ into H_2_O and O_2_ (Qiu et al. 2022; Wang, Song, et al. 2024; Sun et al. 2024; Xie et al. 2024; Feng et al. 2023), the decreased enzyme activities observed in this study suggest that TMX and IMI treatments reduced ROS production, consequently lowering the demand for antioxidant enzyme activity.

PR proteins are extensively induced during the plant defence response (Soheili‐Moghaddam, Mousanejad, et al. 2022; Soheili‐Moghaddam, Nasr‐Esfahani, et al. 2022). IMI‐derived 2‐hydroxy‐CPA analogs potently induce PR1 protein accumulation in *Arabidopsis*, conferring enhanced resistance to powdery mildew (Ford et al. 2010). However, *CaPR1* showed no significant differences between IMI‐primed and control plants at all time points in this study (Figure 4f). TMX can activate jasmonic acid (JA) pathway genes in maize, priming pathogen defence responses (House et al. 2021). The SA‐mediated signalling gene *CaSAR82* (Lee and Hwang 2006), a marker for pathogen‐induced responses, was upregulated in IMI‐pretreated pepper plants compared to controls at 48 hpi, suggesting that IMI may partially activate SA‐dependent defence pathways. *CaDEF1*, a gene involved in MeJA‐mediated signalling, has been shown to participate in plant responses to both pathogen invasion and environmental stress (Shen et al. 2016). *CaDEF1* showed continuous induction from 24 hpi and remained significantly higher expression levels in IMI‐treated pepper leaves compared to controls, suggesting IMI may enhance JA‐mediated signalling, which is associated with resistance to necrotrophic pathogens. The reduced expression of *CaPO1* at 12 and 24 hpi, followed by a pronounced induction at 48 hpi, in IMI‐pretreated plants indicates a temporal response that likely maintains redox equilibrium and restricts excessive cell death.

Conventional strategies for managing *P. capsici* in pepper heavily rely on *R*‐gene‐mediated resistance. Though characterised *R* genes like *CaRGA2*, *CaPOD* and *CaPTI1* confer varying resistance levels (Zhang et al. 2013; Wang et al. 2013; Hong et al. 2008), their efficacy can be limited by the rapid evolution of *P. capsici* physiological races. Intriguingly, *S* genes, which facilitate pathogen infection, show potential for durable resistance (Eckardt 2002; Pavan et al. 2010; Guo et al. 2022). Loss‐of‐function mutations of *S* genes such as *MLO* have demonstrated broad‐spectrum resistance across multiple crops, including pepper (Humphry et al. 2011; Jørgensen 1992; Pavan et al. 2011; Zheng et al. 2013). In pepper, *S* genes such as *Ipcr* and *CaSBP12* have been shown to negatively regulate host defence (Reeves et al. 2013; Zhang et al. 2019), highlighting them as strategic targets for resistance improvement. It has been reported that *NAC45/86* regulates the expression of *NEN1* to *NEN4* by promoting nuclear breakdown in precursor cells of sieve elements in *Arabidopsis* (Furuta et al. 2014; Roszak et al. 2021). Here, we demonstrated that silencing *CaNEN4* enhanced *P. capsici* resistance, whereas *CaNEN4* overexpression intensified necrosis, suggesting that CaNEN4 functions as a pathogen‐exploitable susceptibility factor.

We propose that *CaNEN4* plays a key role in the differentiation of sieve elements, assisting in the programmed cell death that removes the nuclear contents of sieve cells. This is a crucial step for sieve cells to complete their differentiation and become mature sieve elements. The increased *CaNEN4* expression may trigger unnecessary excessive cell death in sieve elements, which could compromise the plant's overall defence capacity. When cell death is excessive, the plant may fail to effectively respond to external pathogen invasion, leading to a decline in disease resistance. In addition, excessive CaNEN4 activity may affect phloem integrity and associated nutrient transport capacity, thereby compromising vascular function during infection. Given that *Phytophthora* pathogens penetrate the sieve element for plant infection, CaNEN4 function disruption may impede pathogen spread by altering host vascular development.

However, how IMI/TMX regulates *CaNEN4* remains unclear. Specifically, direct molecular links between neonicotinoid metabolites and *CaNEN4* transcription have not yet been established. Current evidence supports a host‐mediated priming model in which TMX/IMI metabolites, structurally similar to SA, indirectly suppress *CaNEN4* expression through immune signalling pathways, potentially involving SA–JA signalling and NAC‐mediated transcriptional regulation. Future work should examine CaNEN4 subcellular localization and spatiotemporal expression, and identify its upstream IMI/TMX‐responsive regulators, including relevant transcription factors. To this end, a combination of targeted promoter analysis and promoter‐based screening, such as yeast one‐hybrid library screening followed by chromatin immunoprecipitation‐quantitative PCR validation, would provide an effective strategy to identify transcription factors regulating *CaNEN4*, thereby linking neonicotinoid‐induced priming to phloem differentiation and pathogen spread in sieve elements. Moreover, future stable CRISPR/Cas9‐mediated knockout of *CaNEN4* could elucidate its role in phloem differentiation and its contribution to plant long‐term resistance. From a resistance breeding perspective, targeted silencing of susceptibility genes such as *CaNEN4*, in combination with the expression of classical *R* genes (e.g., Phyto5.2) (Wang et al. 2016), may represent a promising framework to enhance resistance while aiming to maintain balanced growth responses.

Together, this study helps define the pathway linking IMI/TMX, CaNEN4, and plant resistance, and identifies *CaNEN4* as a promising gene target for engineering durable, broad‐spectrum resistance.

## 4 Experimental Procedures

### 4.1 Plant Materials

Sixteen cultivars of *C. annuum* var. *frutescens* (provided by Henan Dingyou Agricultural Technology Co. Ltd., China) were used in this study. Detailed cultivar characteristics are listed in Table S1 and illustrated in Figure S1.

Uniform, healthy seeds were selected and soaked in 55°C water for 30 min, followed by hydration in distilled water for 8 h, and germinated in darkness at 28°C. Germinated seeds were transferred into 72‐cell trays and cultivated in a controlled‐climate chamber (60% relative humidity, 25°C/18°C day/night cycle, 16/8 h photoperiod) for 20 days and then were transplanted into pots for subsequent experiments.

### 4.2 Screening for *P. capsici*‐Resistant Pepper Cultivar

Detached leaves (5th–6th from the bottom) of 40‐day‐old plants were surface sterilised and placed on sterile filter paper. Mycelial plugs (5 mm diameter) from 7‐day‐old *P. capsici* cultures on carrot agar (CA) were inverted onto the abaxial leaf center. Inoculated leaves were incubated under 28°C (16/8 h photoperiod) for 48 h. The mean disease index value of pepper screened against *P. capsici* was based on the methods developed by Bosland and Lindsey (1991).

Disease severity was assessed based on lesion diameter on infected leaves and classified into six grades: grade 0 (0 ≤ diameter < 5 mm), grade 1 (5 ≤ diameter < 10 mm), grade 2 (10 ≤ diameter < 15 mm), grade 3 (15 ≤ diameter < 20 mm), grade 4 (20 ≤ diameter < 25 mm), and grade 5 (25 ≤ diameter < 30 mm). The disease index (DI) was calculated as: [∑(disease grade × number of leaves at that grade)/(highest disease grade × total number of leaves)] × 100. Based on DI values, cultivars were classified into resistance categories: highly resistant (HR, 0–10), resistant (R, 10–30), moderately resistant (MR, 30–50), low resistant (LR, 50–70), and susceptible (S, > 70). Each cultivar was inoculated with nine replicated leaves in three biological replicates.

### 4.3 Exogenous IMI and TMX Treatments on Plants

To evaluate whether IMI and TMX enhance plant resistance, pepper plants with 6–7 true leaves were treated with 1 mM pesticide‐grade IMI or TMX three times within 1 week by gently brushing on the abaxial leaf surface. Control plants were brushed with sterile water at the same intervals. Leaves were collected for *P. capsici* inoculation 1 day after the final treatment.

A split‐plant assay was further conducted to test whether IMI and TMX induce systemic resistance. For each pepper plant, four leaves were selected and divided into two groups. One group was treated with 1 mM IMI or TMX by gentle brushing, while the other group was mock‐treated with sterile water (for pesticide‐grade formulations) or 0.2% DMSO (v/v) (for reagent‐grade formulations). For treatments, pesticide‐grade compounds were dissolved in and diluted with double‐distilled water, and reagent‐grade compounds were dissolved in DMSO and diluted with double distilled waterdH. Treated leaves were labelled ‘+’ (insecticide‐treated) and untreated leaves ‘−’ (solvent‐treated as controls). Treatments were applied three times within 1 week before pathogen inoculation, and leaves were detached for *P. capsici* inoculation 1 day after the final treatment.

### 4.4 TMX and IMI Effect on *P. capsici* Growth and Development

Mycelial plugs (5 mm diameter) of 7‐day‐old *P. capsici* grown on CA were transferred to fresh CA containing serial concentrations (0, 0.25, 0.5, 1, 2.5, 5, 10 and 20 mM) of TMX/IMI. Triplicate plates per treatment were employed and incubated at 28°C in darkness. Colony diameters were measured daily for 7 days. Hyphal ultrastructural features were characterised via stereomicroscopic morphometric analysis. Growth inhibition (%) was calculated as inhibition rate = (control diameter − diameter in treatment)/control diameter × 100%.

### 4.5 Chlorophyll Fluorescence Imaging

Chlorophyll fluorescence parameters were analysed using a FluorCam FC800‐O system (PSI). *P. capsici*‐inoculated leaves were dark‐adapted for 20 min prior to measurement. Real‐time fluorescence images were acquired under controlled low‐light conditions, with optimal focus and illumination intensity.

### 4.6 Histochemical Staining

*Phytophthora capsici*‐inoculated leaves were submerged in 1 mg/mL DAB dissolved in phosphate buffer (10 mM, pH 3.8) or 1 mg/mL NBT dissolved in phosphate buffer (10 mM, pH 7.8), and incubated overnight at room temperature. Following incubation, the respective staining solution was removed. Leaf samples were gently agitated for 15–20 min at 55°C in ethanol to decolourise chlorophyll, then rinsed twice with phosphate buffer and preserved in 70% ethanol for imaging. Three biological replicates were performed for each treatment.

### 4.7 Antioxidant Enzyme Activity Assays

Leaf discs were collected from *P. capsici*‐inoculated sites at 12, 24 and 48 hpi. For each time point, six discs per treatment were pooled, and 0.1 g of tissue was homogenised in 1.8 mL of ice‐cold 0.05 M phosphate buffer (pH 7.0), followed by incubation on ice for 2 h. The homogenate was centrifuged at 1000 *g* for 15 min at 4°C, and the resulting supernatant was stored at −20°C for further analysis. Enzyme activities of superoxide dismutase (SOD, G0101W), catalase (CAT, G0105W) and peroxidase (POD, G0107W) were quantified using commercial assay kits (Geruisi‐bio) following the manufacturer's protocols. All assays were performed with three biological replicates per treatment.

### 4.8 Transcriptomic Sequencing and Data Analysis

Leaves of Cusheng 356 were pretreated three times over 1 week with 1 mM reagent‐grade IMI, with double‐distilled water‐pretreated plants as controls. At 0, 12 and 48 hpi, 14 mm disks centered on inoculation sites were collected (three biological replicates per time point, in total 18 samples), immediately flash‐frozen in liquid nitrogen, and stored at −80°C for transcriptomic sequencing.

Total RNA isolation was performed using a Quick RNA isolation Kit (Huayueyang) according to the manufacturer's protocol. RNA concentration and purity were assessed using a NanoDrop 2000 spectrophotometer (Thermo Scientific), and RNA integrity was evaluated by RNA agarose gel electrophoresis and an Agilent 2100 Bioanalyzer (RNA 6000 Nano Kit; Agilent Technologies). RNA‐seq library construction, Illumina sequencing (paired‐end), and downstream bioinformatics analyses were performed by a commercial service provider (Personalbio, GenesCloud, China).

Libraries were sequenced on the Illumina NovaSeq platform. Raw reads were filtered using fastp (v. 0.22.0) to remove adaptor sequences and low‐quality reads (average quality < Q20). Clean reads were aligned to the reference genome (Pepper Zunla 1 Ref_v1.0) using HISAT2 (v. 2.1.0). Gene‐level read counts were obtained using HTSeq (v. 0.9.1), and fragments per kilobase of transcript per million mapped reads (FPKM) values were calculated for expression visualisation. Differential expression analysis was performed in R using DESeq2 based on raw count matrices with three biological replicates per condition. Genes with log_2_fold change > |1| and FDR‐adjusted *p* value < 0.05 were defined as DEGs. KEGG pathway enrichment was performed using clusterProfiler (v. 4.6.0), with FDR < 0.05 considered significant.

### 4.9 Quantitative Real‐Time PCR

Gene expressions were validated using SYBR Select Master Mix (20 μL reaction: 6 μL RNase‐free water, 1 μL primers, 2 μL cDNA, 10 μL master mix). Cycling parameters were 95°C for 30 s (pre‐denaturation); then 40 cycles of 95°C for 5 s, 60°C for 34 s; then 72°C for 1 min. The primers were listed in Table S3. The 2^−ΔΔ*Ct*T^ method was applied for gene expression analysis. Each reaction was performed with three biological replicates and three technical replicates.

### 4.10 Transient Gene Silencing and Overexpression

The candidate gene's coding region was amplified using homologous arm primers and cloned into the pTRV2 vector. This recombinant pTRV2 vector was subsequently introduced into *Agrobacterium tumefaciens* GV3101. VIGS was conducted when Cusheng L09 pepper seedlings developed fully expanded cotyledons. The *Agrobacterium* culture was cultured to an OD_600_ of 1.0, resuspended in MES buffer, and mixed with an equal volume of TRV1 bacterial suspension. After incubating in darkness for 3–5 h, the mixture was infiltrated into pepper cotyledons. Small holes were first punctured in both cotyledons using a sterile syringe needle, and the suspension was then injected with a 1 mL syringe (needle removed). Seedlings infiltrated with the empty pSN1301 vector served as controls. After a 1‐day dark treatment, the seedlings were continued to be cultured in a controlled‐climate chamber until they developed 6–8 true leaves, at which point they were ready for in vitro leaf inoculation with *P. capsici*.

For overexpression, the coding sequence of *CaNEN4* was amplified and cloned into the plant overexpression vector pSN1301. The recombinant pSN1301 ‐*CaNEN4* vector was then introduced into *A. tumefaciens* GV3101. Cusheng L09 pepper seedlings with 6–8 true leaves were used for pSN1301 ‐*CaNEN4 Agrobacterium* injection, while seedlings infiltrated with the empty pSN1301 vector served as controls. After 1 day of dark incubation followed by 1 day in the growth chamber, the plants were used for *P. capsici* infection.

### 4.11 Statistical Analysis

All experiments were conducted with at least three biological replicates unless otherwise stated, and data are presented as mean ± SD. Data were analysed using GraphPad Prism 9 and SPSS 17.0, and Microsoft Excel 2016 was used for data organisation where needed. For comparisons between two groups, a Student's *t* test was used. For comparisons among multiple groups, one‐way or two‐way ANOVA was applied as appropriate, followed by the least significant difference (LSD) test when ANOVA indicated significance. The significance level was set at *α* = 0.05.

## Author Contributions

Geng Meng, Shujia Wang, Yiheng Hou, Wenqing Li, Shiwei Yang and Kaile Sun conceived and planned the experiments. Yiheng Hou, Wenqing Li, Shiwei Yang, Tianhao Ge, Chenxue Song, Peng Liu, Wenyi Yang, Gonglian Pang carried out the experiments. Yiheng Hou, Wenqing Li, Shiwei Yang contributed to the analysis and interpretation of the results. Geng Meng took the lead in writing the manuscript with the support from Yawen Shen and Kaile Sun. Kaile Sun, Yawen Shen, Chengwei Li, Jianbin Hu and Zhiqi Jia supervised the project, provided critical feedback, and helped shape the research and manuscript. All authors read and approved the final manuscript.

## Funding

This work was supported by National Natural Science Foundation of China, 32301848, 32172575, 31801420. The Key Scientific Research and Development Project of Henan, 252102110184. Henan Provincial Colleges and Universities Youth Key Teacher Project, 2024GGJS028. Henan Provincial Support Program for Science and Technology Innovation Talents in Colleges, 26HASTIT022. Teaching Reform Project of Henan Agricultural University, 2023XJGLX008.

## Conflicts of Interest

The authors declare no conflicts of interest.

## Supporting information


**Figure S1:** The characteristics of 16 pepper cultivars.

**Figure S2:** The growth of *Phytophthora capsici* in growth media supplemented with different concentrations of reagent‐grade imidacloprid (IMI) over a week.

**Figure S3:** The growth of *Phytophthora capsici* in growth media supplemented with different concentrations of reagent‐grade thiamethoxam (TMX) over a week.

**Figure S4:** The real‐time quantitative PCR validation of differentially expressed genes randomly selected from the transcriptomic data.

**Table S1:** List of tested pepper cultivars.

**Table S2:** Disease index and disease resistance grade of 16 pepper samples inoculated with *Phytophthora capsici* at 24, 36 and 48 h post‐inoculation (hpi).

**Table S3:** qPCR primers for measuring differentially expressed genes and PCR primers used for gene cloning.

**Table S4:** Growth inhibition of *Phytophthora capsici* at different imidacloprid (IMI) concentrations.

**Table S5:** Growth inhibition of *Phytophthora capsici* at different thiamethoxam (TMX) concentrations.

## Data Availability

All study data are included in the article and/or Supporting Information.
