# Supplementary figures and images for: Chemical Priming by Neonicotinoids Unveils CaNEN4 as a Susceptibility Gene Against Phytophthora capsici in Pepper
Source: Mol Plant Pathol. 2026 Mar 19;27(3):e70242. doi: 10.1111/mpp.70242 (PMC13097475; doi:10.1111/mpp.70242)

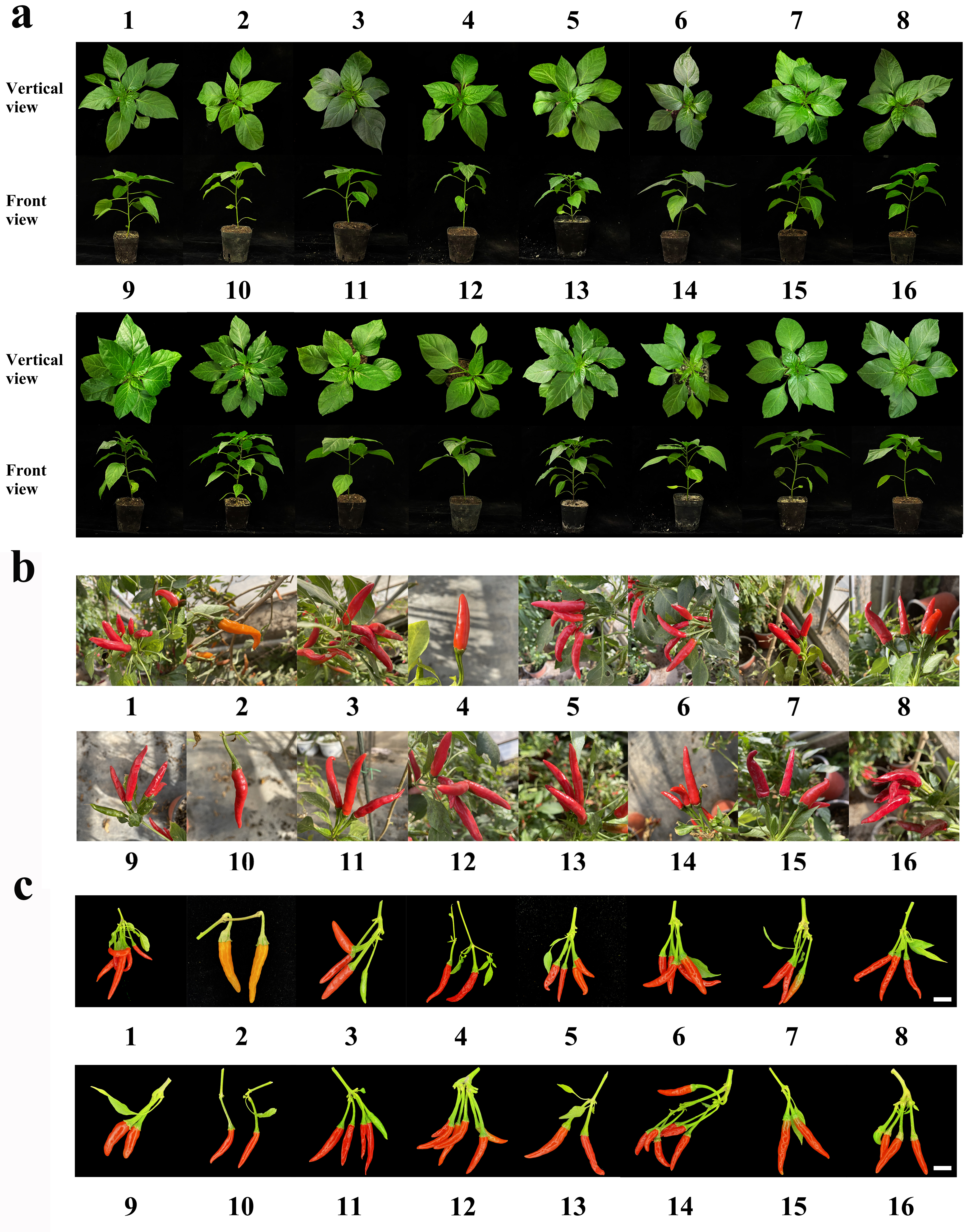

Supplement: Supplementary file 1 — Figure S1: The characteristics of 16 pepper cultivars. [file MPP-27-e70242-s003.jpg]

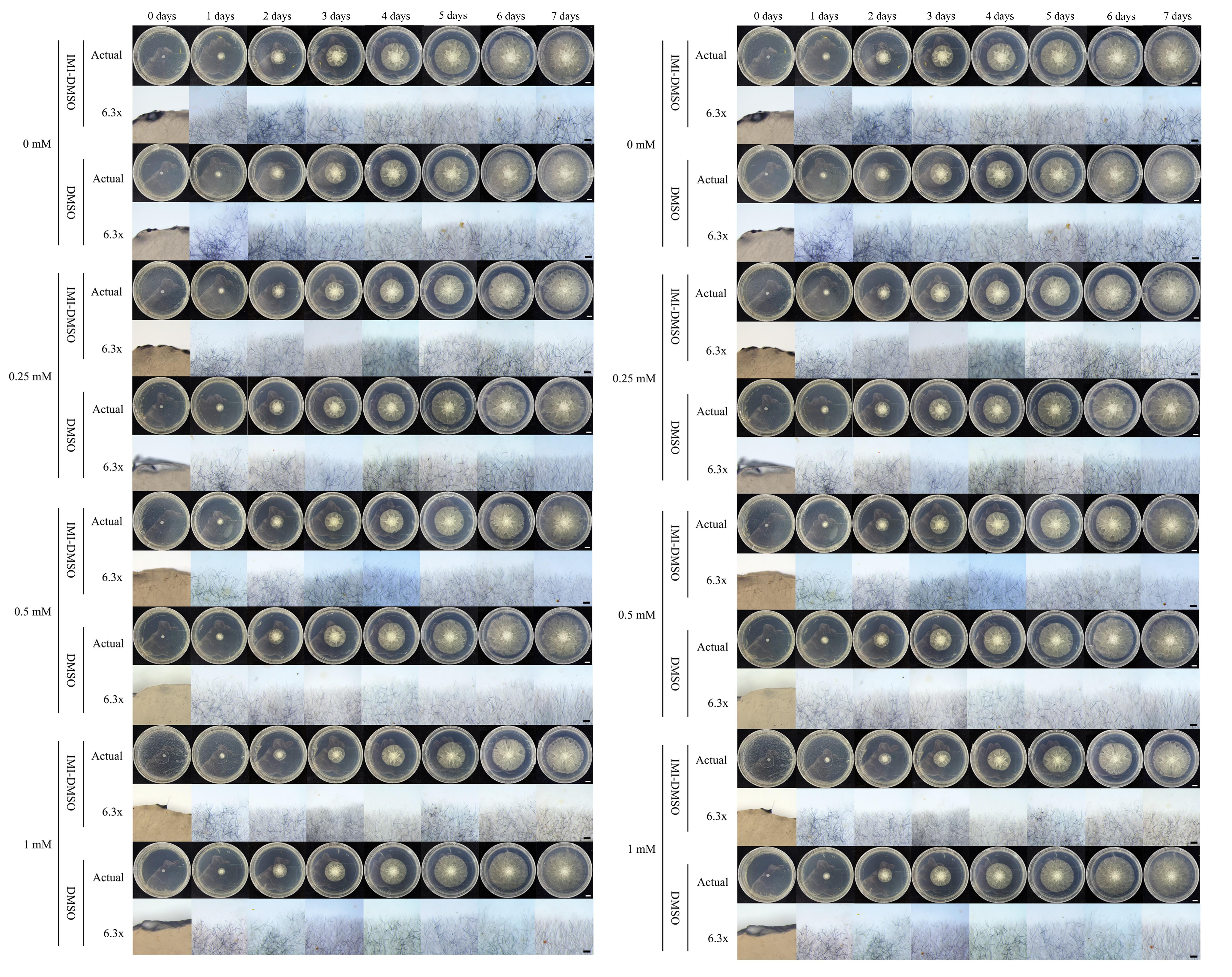

Supplement: Supplementary file 2 — Figure S2: The growth of Phytophthora capsici in growth media supplemented with different concentrations of reagent‐grade imidacloprid (IMI) over a week. [file MPP-27-e70242-s001.jpg]

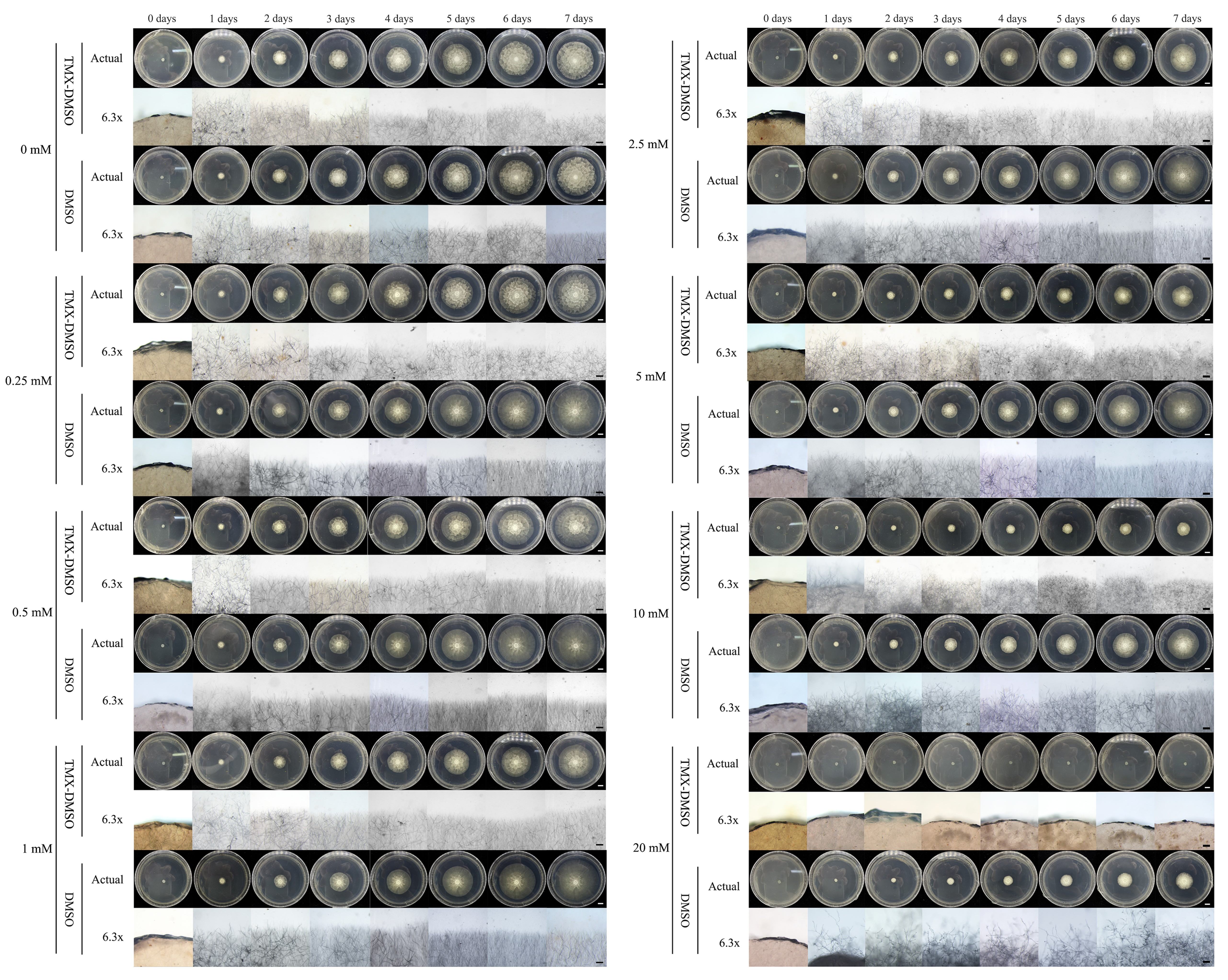

Supplement: Supplementary file 3 — Figure S3: The growth of Phytophthora capsici in growth media supplemented with different concentrations of reagent‐grade thiamethoxam (TMX) over a week. [file MPP-27-e70242-s005.jpg]

Relative expression

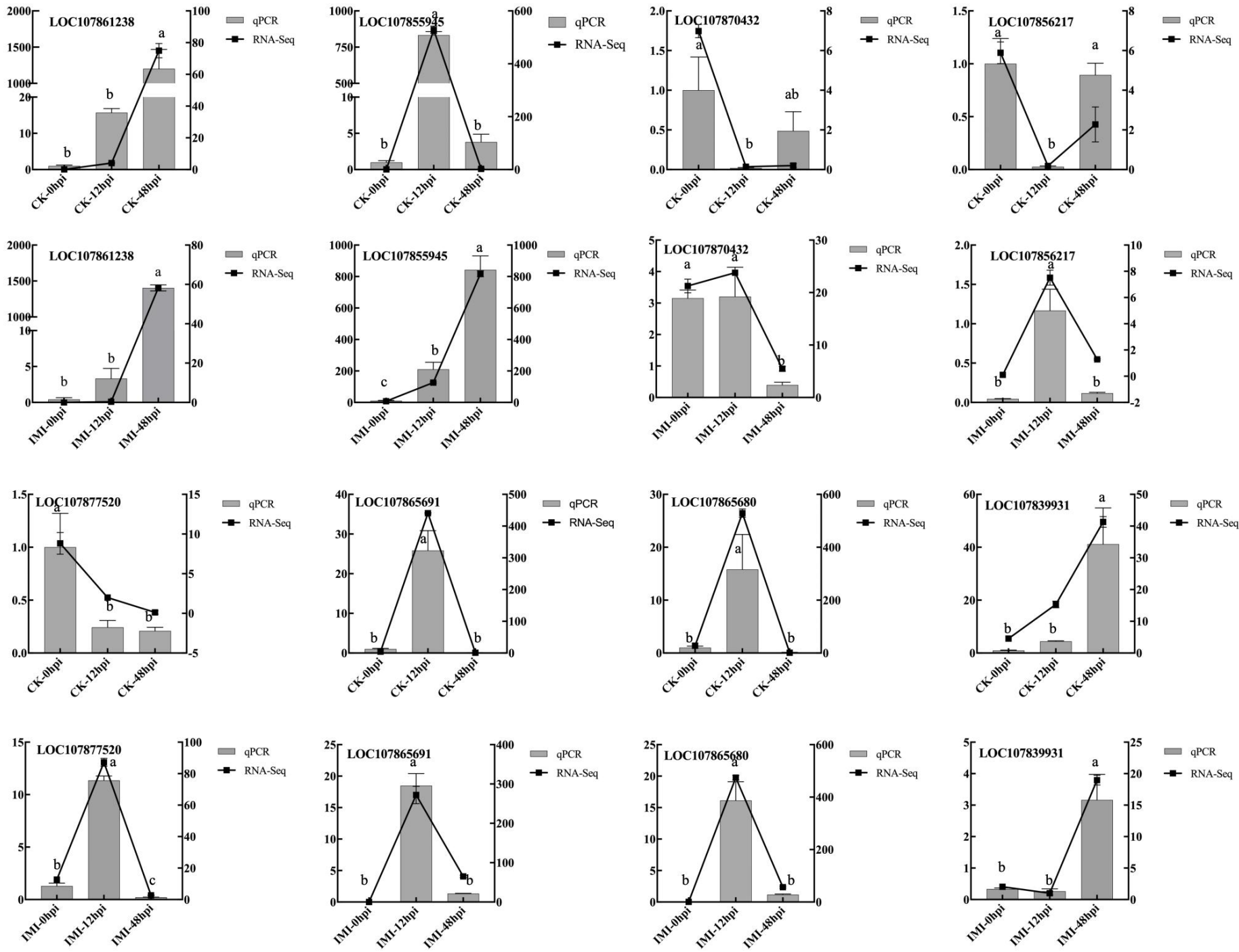

FPKM

Supplement: Supplementary file 4 — Figure S4: The real‐time quantitative PCR validation of differentially expressed genes randomly selected from the transcriptomic data. [file MPP-27-e70242-s002.pdf]
